# Benefits of functional assays in personalized cancer medicine: more than just a proof-of-concept

**DOI:** 10.7150/thno.55954

**Published:** 2021-09-21

**Authors:** Christophe Bounaix Morand du Puch, Mathieu Vanderstraete, Stéphanie Giraud, Christophe Lautrette, Niki Christou, Muriel Mathonnet

**Affiliations:** 1 Oncomedics SAS, 1 avenue d'ESTER, F-87069 Limoges, France.; 2 Centre hospitalier universitaire Dupuytren, Service de chirurgie digestive, générale et endocrinienne, 2 avenue Martin Luther King, F-87042 Limoges, France.; 3 EA3842/CAPTuR (Contrôle de l'Activation cellulaire, Progression Tumorale et Résistance thérapeutique), Faculté de Médecine, 2 rue du Docteur Marcland, F-87025 Limoges cedex, France.

**Keywords:** Cancer, chemosensitivity, CSRA, functional assay, personalized medicine

## Abstract

As complex and heterogeneous diseases, cancers require a more tailored therapeutic management than most pathologies. Recent advances in anticancer drug development, including the immuno-oncology revolution, have been too often plagued by unsatisfying patient response rates and survivals. In reaction to this, cancer care has fully transitioned to the “personalized medicine” concept. Numerous tools are now available tools to better adapt treatments to the profile of each patient. They encompass a large array of diagnostic assays, based on biomarkers relevant to targetable molecular pathways. As a subfamily of such so-called companion diagnostics, chemosensitivity and resistance assays represent an attractive, yet insufficiently understood, approach to individualize treatments. They rely on the assessment of a composite biomarker, the *ex vivo* functional response of cancer cells to drugs, to predict a patient's outcome. Systemic treatments, such as chemotherapies, as well as targeted treatments, whose efficacy cannot be fully predicted yet by other diagnostic tests, may be assessed through these means. The results can provide helpful information to assist clinicians in their decision-making process. We explore here the most advanced functional assays across oncology indications, with an emphasis on tests already displaying a convincing clinical demonstration. We then recapitulate the main technical obstacles faced by researchers and clinicians to produce more accurate, and thus more predictive, models and the recent advances that have been developed to circumvent them. Finally, we summarize the regulatory and quality frameworks surrounding functional assays to ensure their safe and performant clinical implementation. Functional assays are valuable *in vitro* diagnostic tools that already stand beyond the “proof-of-concept” stage. Clinical studies show they have a major role to play by themselves but also in conjunction with molecular diagnostics. They now need a final lift to fully integrate the common armament used against cancers, and thus make their way into the clinical routine.

## Introduction

It is widely accepted that, for diseases with complex and heterogeneous molecular backgrounds such as cancers, “one-size-fits-all” treatment strategies are no longer desirable. This is best illustrated by the unsatisfying response and survival rates observed on unselected patient cohorts for most classes of drugs. To propose and sort patients according to their predicted response to a cure, personalized medicine - or, more accurately, subpopulation medicine - requires reliable tests to stratify and ultimately retain relevant groups of responsive patients. Such tests measure specific response biomarkers in adequate samples. They are formally gathered under the companion diagnostics (CDx) designation 1.

True CDx are currently defined by the US Food and Drug Administration (FDA) as “a medical device, often an *in vitro* device, which provides information that is essential for the safe and effective use of a corresponding drug or biological product [and which] can identify patients who are most likely to benefit from a particular therapeutic product” 2. The European Medicines Agency (EMA) provides a very similar definition 3. Result of the test is usually a sufficient condition for giving or not a linked medication or group of medications 4. Within the CDx family falls a subtype of assays called complementary diagnostics (CoDx). Instead of rigidly directing a patient to a treatment, CoDx rather provide information about the potentially enhanced benefits of receiving a drug. They do not make a specific drug mandatory, though, and a negative result does not disqualify the linked drug. Hence, the main difference between the two types of tests is the freedom of decision for the physicians regarding the choice of treatment for their patient 5.

The history, current role and perspectives of common CDx and CoDx have been extensively reviewed 5-8. So far, such tests have been mostly developed in cancer indications and already possess a crucial role in personalized medicine. A growing number of treatments are now dependant not only on broad diagnosis, but also on the results of tests that identify actionable characteristics (molecular diagnostics). This justifies the requirement for concomitant safety and effectiveness assessments of both the drug candidate and its CDx during development steps 9. Their role will expand in the future: indeed, predictive biomarkers are now integrated early in most drug development programs in oncology 10, with the encouragement of the FDA and a significant impact on drug approval, as exemplified in BIO's Clinical Development Success Rates 2006-2015 11.

The definitions for CDx and CoDx are broad and understood as associating the presence of a specific biomarker (whether a single or a group of mutations, or a protein product) in a patient's body with a specific drug. This refers to the diseased tissue's history 12. Nevertheless, another type of CoDx is frequently overlooked, namely functional assays. These are the mere transposition of preclinical *in vitro* assays, led on selected models to study the response to a drug candidate, to clinically-applicable *ex vivo* assays: indeed, they test on a patient's own cells, upstream of treatment initiation, the arsenal of therapies available for a specific indication. Instead of identifying the roots of a diseased phenotype, they capture the final, and as such clinically relevant, response to a drug produced by the interplay between all biological variables. This outcome acts as a composite, surrogate biomarker for drugs with no known indicator of susceptibility or resistance. Functional assays may hence provide a personalized medicine approach for systemic treatments, including chemotherapies, which have not been clinically associated with single or groups of biomarkers yet.

**Figure 1:**
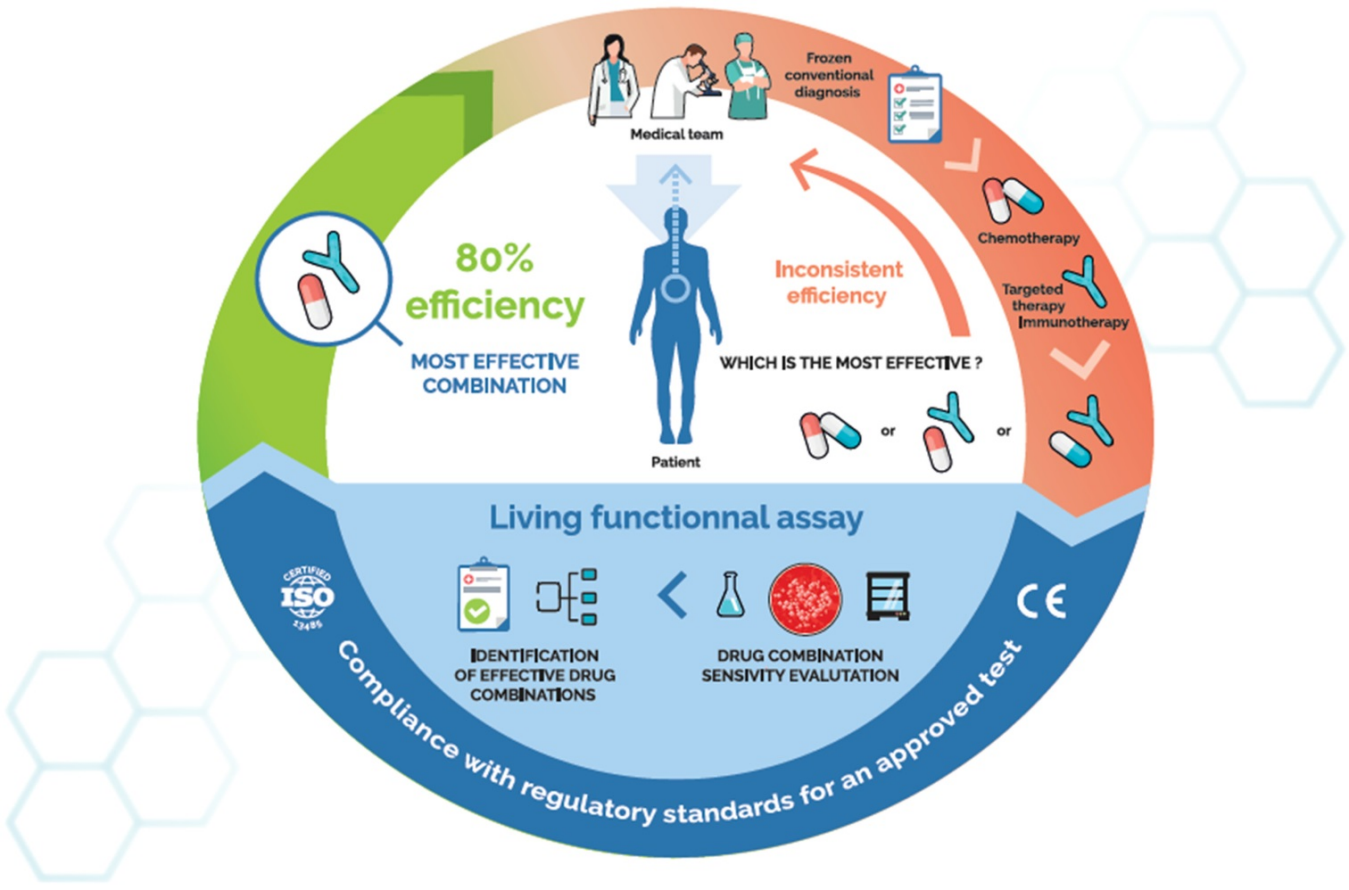
The role of *ex vivo* chemosensitivity and chemoresistance assays (CSRA) in cancer care, a virtuous cycle. Such assays use qualified patient samples and primary culture technologies to directly test the activity of relevant anticancer drugs on a patient's own tumour cells. The resulting chemosensitivity profile is usable by physicians to fine-tune treatments.

Predictive functional assays, especially chemosensitivity and resistance assays (CSRA), have been pursued for several decades 13. They rely on the *ex vivo* modelling of a patient's tumour from pathologically-qualified samples obtained during a medical procedure (Figure 1): diagnosis biopsy, exeresis fragment of primary lesion or metastases, effusion, ascites, blood containing circulating tumour cells… Modelling protocols vary across a wide range, but described workflows share several common features. First, tumour material is processed to two-dimension (2D)/three-dimension (3D) primary cultures retaining the tumour cells' original characteristics. After *in vitro* exposure to treatments of interest, the biological response is analysed through a relevant endpoint to provide a functional profile (chemo-sensitivity or -resistance, DNA repair capacities…), which may mirror that of the original cancer lesions. This profile is ultimately integrated into the clinical decision-making process to individualize the treatment(s) a cancer patient will receive.

Despite long-standing efforts, an efficient protocol still has to obtain recognition by the biomedical community 14, let alone approval by regulatory authorities. The most recent assessment guidelines of CSRA by the American Society of Clinical Oncology (ASCO) came out less than a decade ago 15. They followed a 2004 initial article on the matter 16, and we might infer a similar, updated evaluation might be published within the next 12-24 months. In these two articles, recommendations remained unchanged: “*The use of CRSA to select chemotherapeutic agents for individual patients is not recommended outside of the clinical trial setting. Oncologists should make chemotherapy treatment recommendations on the basis of published reports of clinical trials and a patient's health status and treatment preferences. Because the in vitro analytic strategy has potential importance, participation in clinical trials evaluating these technologies remains a priority*”. This statement enough shows the great potential the ASCO sees in CSRA, albeit they have not fulfilled clinicians' expectations yet.

In this review, we present the most advanced functional assays for treatment individualization in oncology. We then show through a meta-analysis of technical and clinical performances gathered so far that the notion of CSRA is already way beyond “proof-of-concept”. Challenges faced by functional assays, especially in their development along with the expansion of the anticancer drug modes of action (MoA), are presented. Finally, we discuss quality management of the environment of functional assays, as well as regulatory considerations framing their approval, for they provide both hurdles and handrails for the successful implementation of these assays within the clinical routine.

## State-of-the-art: assays showing significant predictive capacities

Pioneer chemosensitivity assays were designed in the late 1970s. They were primarily based on clonogenic properties of tumours and yet showed promising results 17,18. Since then, numerous chemosensitivity assays have been developed to predict *ex vivo* the action of a drug or a combination of drugs on a patient's tumour. Cell culture methods and readouts varied throughout the years, and techniques improved to reach high accuracy levels. In their vast majority, these functional assays follow common steps: (*i*) dissociation of a tumour specimen and isolation of tumour cells, (*ii*) primary cell culture in presence of chemotherapies, (*iii*) assay cell viability/mortality, and (*iv*) data analysis to produce a chemosensitivity profile (Table 1).

Innovative chemosensitivity assays were developed in the late 1980s by three American physicians and scientists, Robert Nagourney, David Kern and Larry Weisenthal. From their work were developed two non-clonogenic assays: the differential staining cytotoxicity assay (DiSC), and the Extreme Drug Resistance Assay (EDR), also known as the Kern assay. The DiSC assay is based on the counting of global cell death among the tumour cell population following drug treatment. Cell death observation relies on the loss of cell membrane integrity, which is observed by dye exclusion using Fast Green. Viable cells are then counterstained with hematoxylin/eosin, then cell mortality is evaluated using a light microscope 19. This technique demands a high level of expertise, since it requires the ability to accurately recognize tumour cells from normal somatic cells. Interestingly, differential staining also applies to dead endothelial cells, allowing the observation of the effect of anti-angiogenics molecules such as the anti-VEGF bevacizumab 20. The EDR assay relies on the measure of cell proliferation by counting the incorporation of ^3^H-thymidine. Following 72 h of drug treatment, cells are incubated with ^3^H-thymidine for another 48 h, allowing its incorporation during S-phase. Results of the assay are categorized as high, intermediate or low drug-resistance, by comparison with untreated controls 21. EDR assay has been commercialized in the USA by Oncotech; however, this CSRA seems no longer available.

Numerous studies investigated the relevance of applying cell metabolism measurement techniques to primary tumour chemosensitivity. The first example is the ATP-Chemotherapy Response Assay (ATP-CRA). This technique relies on total ATP dosage by bioluminescence 22. Tumour tissues harvested following surgical resection or biopsy are cut into small pieces, then cells are separated by enzymatic digestion. A given number of cells are cultured in presence of drugs, then the amounts of ATP are measured using a luminometer. Any drug impairing cell growth or proliferation will ultimately decrease the total amount of ATP within cells. From these results, a cell death rate is calculated. The high sensitivity of ATP bioluminescence allows to miniaturize the assay, which necessitates little tumour material.

Another cell metabolism assay designed for chemosensitivity assessment purpose is MTT. This technique has been extensively used for the development of the Histoculture Drug Response Assay (HDRA). Unlike ATP-CRA, HDRA is based on the culture of small pieces of the tumour (1-2 mm) on collagen-coated matrices 23. Fragments are treated with chemotherapies for 48 to 96 h, then cell viability is evaluated. Another system named ITRA (Integrative Tumor Response Assay) was developed to determine the efficacy of second line treatments 24,25. It consists in two successive HDRAs, the latter being performed on cells that survived the first round of chemotherapy. Another technique, called Collagen gel Droplet embedded culture Drug Sensitivity Test (CD-DST) has been widely developed. The CD-DST general concept relies on the embedding of tumour cells in collagen droplets to mimic the *in vivo* situation 26. One advantage of this method is that collagen droplets gather tumour cells as well as non-tumoral cells and ECM components. Following droplet formation, cells are cultured in presence of anticancer drugs, and cell viability is assessed by estimating neutral red uptake and by measured growth inhibition rate (IR). IR is expressed as T/C ratio, with T being the optical density of treated collagen droplets, and C being that of the control group. *In vitro* drug sensitivity is then determined by implementing a growth inhibition rate threshold, which is mostly above 50%.

Although less developed, several other academic initiatives are worth mentioning, notably Fluorescent Cytoprint (FCA) and Sulforhodamine B (SRB) assays. FCA measures the activity of cytosolic esterases, which convert non-fluorescent fluorescein monoacetate into fluorescein. Small tumour fragments, named micro-organs, are cultured on collagen-coated metal grids. Drug-induced cytotoxicity is measured by comparing cytoprints, *i.e*. fluorescent microscopy pictures, before and after drug treatment 27. Sulforhodamine B assay is a colorimetric assay that consists in quantifying the total amount of proteins, which reflects total cell number. To our knowledge, only two publications mentioned this assay as a putative CSRA 28,29.

Apart from these academic initiatives, several proprietary assays were also developed by biotechnology companies. Below are detailed five of the most advanced techniques:

- The MicroCulture-Kinetic assay (MiCK) is a drug-induced apoptosis assay. The history of this assay relies on the observation that most chemotherapies induce cell death *via* apoptosis 30. Membrane blebbing and nucleus condensation are hallmarks of apoptosis: as such, they participate in a rise of optical density. Then the principle of MiCK assay is to measure OD_600_ following drug treatment 31. Using a proprietary algorithm, the MiCK assay converts OD changes into Kinetic Units (KU), which indicate tumour chemosensitivity. First described in 1994, the MiCK assay is a precursor CSRA documented with numerous technical and clinical studies. Initially designed for leukaemia cells, the MiCK assay was later applied on solid tumour samples, including ovary, breast, lung and endometrium 32-34. It is now commercialized by Pieran Biosciences as ChemoINTEL™;
- The ChemoID^®^ assay has been recently commercialized by US-based company Cordgenics. It is atypical since it aims at providing separate bulk tumour cell and cancer stem-like cell (CSC) responses to chemotherapies. Procedures include the following steps: tumour dissociation, CSC enrichment in a bioreactor, cell sorting by flow cytometry, chemotherapy treatment, and finally a WST-8 cell proliferation assay 35. This innovative technology looks to overcome CSC chemotherapy resistance occurring in some cancers, and to prevent recurrence. Randomized, assay-directed clinical trials are currently ongoing against glioblastoma and epithelial ovarian cancer, with overall response rate as primary outcome measure (ClinicalTrials.gov numbers: NCT03632798, NCT03949283 and NCT03632798);
- The ChemoFX^®^ assay has been developed by the US company Helomics and is dedicated to gynaecological cancers 36. Its endpoint is total DNA quantification. Global procedure includes primary culture of 1 mm^3^ tumour fragments until confluency, then trypsinization and subculture into 384-well plates with drugs. Serial dilutions are tested and, following a 72-h treatment, cells are stained with DAPI then counted by fluorescent microscopy. Tumour response is evaluated by measuring the area under the curve.
- The CANscript^®^ technology has been developed by Indian company Mitra Biotech. This technique recreates the native tumour environment by culturing thin tumour explants into a 3D matrix, in presence of autologous serum 37. Prediction of clinical outcomes is achieved by combining several readouts such as viability, proliferation and apoptosis using a machine learning proprietary algorithm;
- The Oncogramme^®^ has been developed by the French company Oncomedics to which belong/belonged some of the authors of this review. It consists in the measurement of drug-induced cellular mortality using fluorescence microscopy. Its main advantage lies in the use of a fully standardized, serum-free cell culture medium, allowing both optimal reliability and high culture success rates 38-40. Moreover, the Oncogramme^®^ was designed to measure the effect of drug combinations routinely used in cancer patient care, such as FOLFOX or FOLFIRI. The Oncogramme^®^ was originally developed against metastatic colon cancer (ClinicalTrials.gov number: NCT02305368), and its use in breast and ovary cancers is currently under investigation (ClinicalTrials.gov number: NCT02910622). The Oncogramme^®^ is, to our knowledge, the only CE-marked functional assay, allowing its distribution within the European Union. A randomized, assay-directed phase 3 clinical trial is currently ongoing to evaluate its capacity to improve 1-year progression free survival (PFS) of metastatic colon cancer patients (ClinicalTrials.gov number: NCT03133273).

## Benefits brought by functional assays across indications: a meta-analysis

### Criteria used to evaluate CSRA technical performances and results

Numerous proof-of-concept studies have been performed on a wide panel of malignant pathologies using methods described above. Table 2 and Table 3 summarize studies in which clinical and technical performances have been evaluated.

A total of 42 studies have been included, from 1991 to 2019. Clinical studies older than 1990 and/or related to clonogenic assays were deliberately omitted.

First interesting observation is the high primary culture success rate obtained in most studies. It ranges from 43.8% to 98.8%, with a mean of 86.6%. Of note, the lower limit of this range, obtained on lung cancer 47, was the only value below 72.7% among the 28 we report here. When excluded, the mean rises to 88.2%, better accounting for the success rate of primary cultures. This clearly demonstrates the ability of all these methods to culture primary tumour cells from tumour explants. Primary cell culture is often optimized using serum-containing media, which favour cell proliferation, but do not allow assay standardization. Notably, ATP-CRA and HDRA protocols usually add 5 to 20% serum to culture media 47,70. Noteworthy, this hindrance has been overcome in several techniques such as the Oncogramme^®^, without affecting success rate 39.

In the context of CSRA, sensitivity represents a key parameter to study, since it measures the proportion of responders properly identified as such by the assay 97. In other words, sensitivity is an index of prediction efficacy. Sensitivities calculated in studies and listed in Table 2 vary from 44.4% to 100%, with a median of 98.0%. No method shows any greater efficiency, as mean sensitivities for all techniques are all above 80% 9. Noticeably, the ITRA method has lower sensitivity percentages. However, this exploratory technique aims at evaluating tumour response to second-line agents, and therefore could hardly be compared with other CSRA. As a result of such good sensitivity indexes, positive predictive value (PPV), which measures the proportion of accurate predictions among all positive calls, is also high and reaches a median of 83.0% (Table 2). This high percentage emphasizes the global precision of chemosensitivity assays.

Opposite to sensitivity, specificity represents the percentage of true negative, *i.e.* the proportion of non-responders among the population found as such by the assay. In studies listed in Table 2, specificity is significantly lower than sensitivity, but still reaches 72.7%, with percentages varying from 18.2 to 100%. In that context, specificity actually corresponds to chemoresistance measurement, which is not the primary objective of chemosensitivity assays. Besides, dedicated assays have been developed to measure chemoresistance, the most advanced one being the Extreme Drug Resistance Assay (EDRA) 21,98,99. Negative predictive value (NPV), the index of false negatives among all negative calls, has a median value of 82.9%, suggesting that in most cases CSRA are also able to accurately identify non-responding patients. Finally, accuracy (measured as the percentage of true positive and true negative patients among total population) represents the best index of CSRA effectiveness, as it measures the probability of a correct prediction. In this meta-analysis, accuracy ranges from 44.4% to 94.4% and reaches a median value of 77.8%.

Taken together, these results demonstrate the accuracy of chemosensitivity assays, with an ability to predict positive outcomes of around 90%. Despite being high, prediction of non-responders is still perfectible in most cases, as specificity is almost systematically lower than sensitivity. However, results must be taken with care, since many of the abovementioned studies were conducted using heterogenous samples, *i.e.* which already underwent chemotherapeutic treatments, or from diverse histologic grades. Because of this lack of consistency, one can hardly determine whether one chemosensitivity assay gives better results than another. To our knowledge, only few comparative studies were performed on the same tumour material. In ovarian cancer, it has been shown that DiSC, ATP tumour chemosensitivity and MTT assays correlate well 100. Further comparative studies will be needed to answer about this question.

## Overview of CSRA clinical performances

To ensure the reliability of CSRA, technical performances must be linked to clinical benefits. Among 27 clinical studies listed in our meta-analysis (Table 3), only two of them failed to find significant benefits for the patients 88,93. Most of these studies were retrospective evaluations and allowed to correlate chemo-responses to clinical outcomes. In various studies, patients treated with a regimen for which they have been found sensitive showed better clinical outcomes in terms of PFS, time to progression (TTP) or overall survival (OS). As an example, patients with advanced ovarian cancer found as paclitaxel- and carboplatin-sensitive using HDRA showed a significantly longer PFS than patients categorized as resistant (34 *vs.* 16 months, p = 0.025) 95. These encouraging results were obtained in studies covering a wide range of solid malignancies including breast, colon, lung or gastric cancers.

So far, seven studies were conducted in a prospective manner, with treatments being guided by CSRA results. Again, patients who benefited from CSRA showed better clinical outcomes. OS was statistically improved in one study (14.6 *vs.* 7.4 months, p = 0.041) 90. In addition, several other studies put into evidence longer PFS 91, time to progression 77 or response rates 33,46,77,81,82,91. To our knowledge, only two randomized study investigated the contribution of CSRA-guided chemotherapy 82,93. Some other blinded, randomized studies are currently ongoing in stage IV colon cancer (the Oncogramme^®^, NCT03133273), glioblastoma (ChemoID^®^, NCT03632135) and ovarian cancer (ChemoID^®^, NCT03632798).

Besides clinical cohort studies, case reports also highlighted the usefulness of chemosensitivity testing before choosing a chemotherapy 101-108. Among others, ATP-based and CD-DST assays were used to help treatment decision for rare pathologies such as colorectal choriocarcinoma 101, Stewart-Treves syndrome 106, or parathyroid carcinoma 108.

Taken together, this meta-analysis clearly demonstrates the efficacy and usefulness of CSRA in various cancer types. Beyond satisfying technical performances, clinical studies also showed that the use of CSRA could lead to clinical benefits for patients. However, as urged by the ASCO, this tendency must be confirmed with more solid interventional, randomized studies, with patients being treated according to the results of the assays.

## Expanding the field of functional assays

In the previous sections, we discussed the most advanced functional assays, having reached a high degree of prediction through clinical evidence. Each assay has its own advantages and limitations, leaving room for substantial improvement in *ex vivo* modelling and measurement of functional response to an ever-expending array of drug classes. Several parameters should be taken into consideration for a continuous adaptation to biology and medical practice. In this section, we pinpoint those we identified as the most relevant. In addition, we discuss other technologies dedicated to treatment response prediction, with varying degrees of development and clinical implementation.

Accurate cancer models are more crucial than ever, both at the drug development and clinical practice levels, as illustrated by the despised role of historical cell lines 109. Heterogeneity and microenvironment influence are main targets in the development of relevant *ex vivo* models. Intrinsic heterogeneity is one of the characteristics of cancer, deriving from both genomic instability, one of its so-called hallmarks, and immunoedition 110-112. A first degree of tumour heterogeneity is reflected in metastasis. Indeed, advanced solid tumours spread through metastasis from a subset of cells displaying a more aggressive and mobile phenotype. Throughout the course of carcinogenesis, it is hypothesized these resistant cells progressively overcome other tumour cells, notably through Darwinian selection induced by selection pressure 113, immunoedition 114 and tumour repopulation following chemoradiation treatment 115. They then produce distant lesions whose characteristics may greatly differ from the primary lesion. The purpose of chemotherapy in adjuvant setting is to control the primary lesion and avoid the occurrence of metastases. In the neo-adjuvant setting, its aim is to reduce the size of metastases and bring them to resecability. For advanced/metastatic solid cancers, one may thus wonder whether it is appropriate to target only one lesion for chemosensitivity assessment, especially if it is the primary tumour. Chemosensitivity comparison between primary and distant lesions has been investigated. Results actually suggest different profiles, with metastases being frequently more resistant to chemotherapy than primary lesions 116-118. However, the true clinical impact of this difference remains to be established: a randomized study on CRC using ATP-CRA showed that treatment selection upon chemosensitivity profiles deduced from primary lesions still increased liver metastasis resecability rate 82. It appears the best scenario would be for clinicians to receive comprehensive information about the highest possible number of a patient's lesions to finely tune their therapeutic approach. This could lead to the destruction of all tumour cells, independently of their degree of aggressiveness. However, in most cases, every lesion will not be accessible for sampling. Sampling being selective in nature, there is a non-negligible probability to miss during this procedure a tumour zone containing more aggressive subclones 111. This sampling bias can occur both at the tumour resection and histopathological analysis steps. In addition, if several samples must be tested for each patient, this may significantly raise the complexity and costs of the whole procedure, making it economically less relevant. Fine cost calculations should be performed once the final test format is set, and appropriate resources mobilized accordingly to ensure full accessibility to patients. As metastases are the main target of chemotherapy, their functional analysis should be prioritized over primary lesions. Also, despite arguably being the most crucial switch in anticancer therapy, tumour invasion and metastasis blockades has received less attention than cytotoxic therapies 119. Future investigations will hopefully demonstrate whether treatment individualization may also be envisioned in that area.

Metastasis spreading through rare, mobile cells illustrates the importance of capturing functional response at the single cell level, instead of basing recommendations on a global response of mixed cell populations. Current CSRA do not differentiate between cancer cell subpopulations. Consequently, they are not able to identify treatment-resistant cells, usually present in very low number, before they generate disease recurrence. Most assays select tumour cells from the large number of cells extracted from sampled primary or distant lesions, whether by differential centrifugation, functionalized microbeads, FACS or selective culture conditions. These assays hence rely on a more homogenous subpopulation to derive clinically useful sensitivity profiles. However, rarer, more aggressive cells still end up lost within the dynamic range. Either label-free cell sorting technologies 120 or co-staining protocols coupled to image analysis and detection algorithms may improve the specific detection of rare cells at any relevant step of the CSRA workflow. Circulating tumour cells remain easily accessible and represent a target of choice in that regard. Their chemosensitivity prediction role has proven possible on various cancer samples from molecular signatures 121. Their transferability to *ex vivo* assays, however, may require sufficient expansion of this rare material, with spheroids as the final testable product 122,123. Additionally, effusion-derived cancer cells, accessible and more abundant, have also proven another useful source of material for chemosensitivity prediction in a large array of cancers 124.

Another bias that hampers representativeness of *ex vivo* models results from the molecular drift that may occur during the primary culture phase. This has indeed been a concern for patient-derived xenografts (PDX) models 125, 126 discussed later in this section, although overall stability throughout passages has actually been demonstrated in colon PDX collections 127. CSRA used in a clinical environment aim for a reduced turnaround time for practicality reasons. Consequently, short-time primary culture, with very limited number of passages, should limit this effect and preserve representativeness 128. Mid- or high-throughput molecular biology technologies such as qPCR-based assays (already implemented into the clinical setting for diagnosis purpose) or next-generation sequencing methods (making their way through the clinical laboratory), combined with pathology-specific gene panels, may help ensuring the most relevant molecular alterations are maintained in the cultivated cells of a specific model 129. Once again for practical reasons and reduced costs, such approaches can be utilized at the research and development stage of a CSRA, to establish the model's quality. The continuous clinical implementation of advanced molecular technologies may later allow their direct use during clinical implementation of the CSRA, as a quality control tool.

Tumour heterogeneity is also reflected in tumour microenvironment (TME). Two-dimension (2D) models obtained from dissociation of tumour tissue have been favoured so far. Obviously, such models are not comprehensively representative of tumour contexture, lacking key elements from the TME that cannot be fully compensated for with specifically formulated culture media. 3D models receive more and more attention to study the impact of drugs on tumour growth, neo-angiogenesis, or interaction between immune cells and tumours 130,131. 2D models do not preserve tumour architecture, both at the tissue and cellular levels, nor do they systematically maintain the expression of relevant signalling intermediates 132; they may also alter tumour cell proliferation compared to their patient counterpart 133. 3D models, on the other hand, are thought to better recapitulate actual pharmacokinetics, with gradients of drug accessibility within the 3D structure, and an inner core that might stay safe from cytotoxic activity. Such models encompass a large array of technical approaches: tumour cells/fibroblast cocultures with direct contact (examples reviewed in 130), spheroids derived from either single cell or multicellular structures 134, organoids 135, tissue fragments 136. This list can be extended with more recent bio-printing techniques: they use layer-by-layer deposition of bio-inks to combine tissue spheroids or cell pellets with de-cellularized extra cellular matrix; effective vascularization through a computer-aided pre-designed structure allows generating viable 3D constructs 137, 138. 3D models also increase the diversity of measurable endpoints in space and time, from metabolic activity to biomarker immunodetection 131. Some assays have been brought to *ex vivo*/*in vivo* comparison to study the predictive capacities of the model, a few examples of which follow. First, short-term culture of tumour fragments on poly-2-hydroxyethylmetacrylate (PolyHEMA) was used to investigate the chemosensitivity of various patient samples, including liver metastases, to irinotecan active metabolite SN-38 139. This coating produced 3D nodules, while preventing fibroblast invasion during culture. A proliferation index was measured on nodule sections. Comparison with response evaluation criteria in solid tumours (RECIST) 140 measured in a small patient subset suggested a trend, albeit statistically insignificant, towards effective response prediction to SN-38 for this model. It does not seem to have been pursued further yet to evaluate clinical significance. A more ambitious study demonstrated the representativity of patient-derived organoid models of CRC and gastroesophageal cancers 141. It was shown that spatiotemporal molecular and expression heterogeneity was preserved for different lesion sites and over treatment course; in addition, excellent chemosensitivity performances were observed. Exploiting similar models, the *TUMOROID* clinical trial studied the capacity of a CRC patient-derived tumour organoid-based assay to identify non responders to first- and second-line chemotherapy 142. While being hampered by only a 63% culture success rate, it demonstrated excellent predictivity, although for irinotecan-based therapies only. In CRC-induced peritoneal carcinomatosis, chemotherapy is a major instrument at various level of disease management 143, notably intraperitoneal chemotherapy associated to hyperthermia (HIPEC) and pressurized intraperitoneal aerosol chemotherapy (PIPAC). Organoids generated from CRC patient-derived xenograft (PDX) models have proven effective to study the efficacy against peritoneal metastases of chemotherapeutic protocols involving HIPEC 144. Building on these seemingly relevant models from Fanny Jaulin's laboratory, a clinical study termed ORGANOTREAT-01 should start soon, investigating the predictive capacity of organoids from digestive cancer samples against a large drug panel. All these models involve 3D structures obtained from non-adherent culture conditions, which, contrary to gel-like matrices, allow better addition and washout of drugs. However, they do not accurately mimic the *in vivo* drug bioavailability influenced by, among other factors, administration route, plasma clearance, extracellular matrix and cancer-associated fibroblasts (CAF). A thorough reconstitution of TME is illustrated by the CANscript^TM^ platform, which uses explants cultivated within a tumour grade-matched protein matrix supplemented with autologous patient serum 37,145,146. Still, such assays complexify the whole procedure, especially when autologous supporting material (serum, fibroblasts) is required. This may negatively influence time-to-results and costs, thus restricting test execution to specialized laboratories.

Desirable characteristics to improve models include miniaturization, tight control of culture conditions over time, throughput enhancement, and study of orthogonal functional response parameters 37. Borrowing to other “hard sciences”, more complex culture systems couple these characteristics with targeted imaging or electromagnetic endpoints: technologies such as microfluidics 147 and organ-on-a-chip, also termed as “microscale cell culture analogues” 148, are used to measure chemosensitivity. Numerous tumours can be effectively cultured, such as lung, bone marrow, brain, breast, urinary system (kidney, bladder and prostate), intestine and liver. Co-culturing of multi-tissue types in tumour-on-a-chip systems, and specifically heart-liver-intestine co-culture 132, which allow studying interdependdent effects of multiple miniaturized organs, offer the most realistic models to recapitulate the tumour *in vivo*-like microenvironment. Label-free, non-destructive biophysical treatment sensitivity biomarkers are also investigated, hence mass accumulation rates determined at the single-cell level 149,150, or shifts in impedance spectra of melanoma fragments measured by impedance spectroscopy 151. Applicability to every cancer, being “solid” or “liquid”, of all these modelling technologies, whether 3D or biophysical, remains to be demonstrated 142.

Besides accurate tumour modelling, to be predictive an assay should measure: (i) how efficiently a drug induces cancer cell killing, as it is the foundation of tumour shrinkage; and (ii) which cancer cells are killed among a heterogeneous subpopulation; as previously stated, the latter is particularly important to predict recurrence. Chemoradiation therapy induces cell death through a large variety of mechanisms 152,153. A true measure of cell death, including all its forms and without losing information due to assay conditions 154, should best predict the effect of a cytotoxic drug 153,155,156. Future assays should be more adaptable to specific drug MoA. A solid biomarker hypothesis is required for trustable CDx. It appears less necessary in the case of functional assays, provided the chosen endpoint is clinically meaningful and appropriately measured. Standardization is currently an aim, especially when developing cost- and time-effective assays. This, however, leads to the oversimplification of *ex vivo* models we previously emphasized, making it challenging to reproduce pharmacokinetics of drug exposure *in vitro*. Depending on the drug's MoA, it may rather be required to finely tune its conditions to fully study its effects, then predict a clinical response. This could be done by controlling total duration of assay and specific duration of drug exposure, as well as measuring cell response at different time points and concentrations, in presence of the drug or after a recovery time. Microfluidics-based technologies, previously cited, may bring the required plasticity.

The essential and ambiguous role of immune contexture in modulating both clonal evolution patterns of cancer through immunoedition 114 and patient's prognosis 157,158 has now been clearly demonstrated. The therapeutic revolution brought by immuno-oncology drugs, especially immune checkpoints inhibitors (ICI), has nevertheless produced inconsistent clinical benefits. Outcome has been linked to the extent of cancer-induced immune priming, which in turn may be predicted by biomarkers, *e.g.* tumour mutation burden (TMB), PD-1/PDL-1, and tumour-infiltrating lymphocytes (TIL). Percentages of response are variable and, to some extent, fail to be accurately predicted by biomarkers 159. Primary culture of cancer cells to produce a relevant *ex vivo* model is challenging; adding another level of complexity, that is to say coculturing cancer cells with autologous immune cells and measuring a response, is even more challenging. Yet, several functional assays are being developed 160, using various readouts. A so-called gold standard technique is the chromium 51 release assay, which has been used for decades 161. Easier to implement, biophysical parameters such as real-time cellular impedance can be converted into cytolysis measurement to study the *in vitro* activity of most immunotherapy drug classes and immune effector cells 162. Closer to bedside, changes in immune activity against tumour cells have been measured in melanoma explant models through the histological identification of relevant actors and subsequent measurement of both immune infiltration and inter-cell distance, pre- and post-treatment with anti-PD-1 nivolumab 153. Patient-specific responses to ICI have also been observed using the CANscript^TM^ platform 163. Regarding the specific targets PD-1/PD-L1, the ability of CoDx to predict tumour sensibility to immunotherapies has been inconsistent so far, especially due to the variable expression of these markers in space and time 7. It may be expected such difficulty will also be encountered in *ex vivo* sensitivity assays aiming at predicting tumour response to ICI targeting this pair, as well as, more generally, any other non-static target with a fluctuating and microenvironment-dependant presence.

Another category of anticancer drugs, anti-angiogenics, are challenging to test using conventional 2D/3D *ex vivo* models, especially because they would require a longer time to allow tumour model vascularization. Preclinical models exist but are not easily amenable to the clinic 152, and their value would probably be inferior to that of functional assays dedicated to drugs that have a direct effect on tumour cells. A surrogate approach investigated the neutralizing effect of bevacizumab on circulating VEGF using a VEGF-dependent cell line subjected to sera of cancer patients treated with bevacizumab suggested potential to predict clinical benefit 164. Chick chorioallantoic membrane (CAM) assay has also established as an interesting model to study tumorigenesis and antiangiogenic treatment effects 165,166. This approach, easier to implement than PDX, may thus be considered as well for tumour response prediction studies. The embryonated egg is a highly vascularized, nutrients- and growth factors-enriched environment, in which cells from primary tumours or cell lines can be easily grafted. This *in vivo*-model is easier, time- and cost-competitive to generate as compared to mouse models. The whole process allows obtaining usable tumours within 10-13 days of grafting; it thus appears suitable for clinical applications. CAM has hence been used for testing chemosensitivity or chemotoxicity 167. Its accessibility allows performing topical and intravenous administration of anticancer drugs, as well as determining optimal irradiation conditions for photodynamics therapies in multiple tumour samples 168. More recently, tumour grafting models and functional testing were developed on zebrafish models: Fior et al. showed that xenografts in zebrafish larvae have enough resolution to measure interpatient and intrapatient heterogeneity in chemotherapy response in 4 days 169, and to predict tumour response to radiotherapy 170.

DNA repair capacities of cancer cells can be studied *in vitro* from cell extracts and represent a promising tool to predict response to drugs targeting DNA damage response pathways 171, especially PARP inhibitors 172. In addition, microsatellite instability (MSI), TMB and neoantigen load are the consequences of DNA repair impairment. As such, they play an important role in clinical response to ICI 159, whose activity might also be predicted by DNA repair functional assays. *In vitro* detection of DNA damage may be approached through analytical chemistry, molecular or immunological methodologies 173. So far, this has been especially beneficial to predict radiotherapy-induced adverse events from peripheral blood lymphocytes (PBL) 174. Nevertheless, the Comet assay, commonly employed in genotoxicity studies to measure lethal DNA strand breaks, was used to assess radiosensitivity in bladder cancer 175 and sensitivity to topoisomerase I inhibitor irinotecan in CRC 176. Interestingly, in the latter work, predictivity was based on *ex vivo* results obtained on more easily accessible PBL rather than directly on tumour cells, suggesting a surrogate target for measuring specific anticancer activity. The Comet assay has also proven useful to measure alkylation and addition products generated by chemotherapies, suggesting a transferability to CSRA 177. A recent clinical study showed no advantage of either irinotecan- or oxaliplatin-based regimen against mismatch repair-deficient/MSI metastatic CRC 178. It would be interesting to investigate with a CSRA if this absence of difference, visible within an all-comer cohort, is also reflected at the individual level.

For drug discovery purposes, PDX-based* in vivo* models appear scientifically sound to preserve the tumour's characteristics, recreate its environment and study the response of a whole sample instead of individualized cells or clonal groups 141,179,180. However, several hurdles may impair their implementation into the clinical setting for treatment individualization: (i) availability of sufficiently humanized animals; (ii) low throughput; (iii) unpredictable graft take rate; (iv) heterogeneous growth rate, resulting in variable and potentially unacceptable time-to-results; (v) model drift through sample fragmentation and passages 181; (vi) costs, sanitary and ethical issues associated with the use of laboratory animals. In addition, one can expect the host would introduce biases altering the sensitivity profile of the grafted sample 182. Chemosensitivity prediction through the use of PDX models has been tried on heterogeneous cohorts of patients with solid cancers 183,184. Yet, because of the previously listed drawbacks, especially take rate and propagation time, we question their current applicability within the clinical setting.

Because of their demonstrated predictive capacities on patient-derives models, functional assays are interesting tools for drug preclinical validation. Some contract research organisations (CRO) rely on such business model. Following *in vitro* assessment of a drug's MoA, functional assays allow preparing *in vivo* and first-in human studies by identifying and/or confirming disease subtypes most susceptible to respond to the drug candidate. This additional data investigating the biological hypothesis of a drug might reinforce the Investigational New Drug (IND) dossier. Additionally, it is worth noting that functional assays have another role to play in drug clinical development. Since they rely on a specific type of biomarker, functional assays may also help shaping clinical trials, much like CDx are doing with umbrella and basket trials 185. However, similarly to biomarkers approved across several indication, functional assays are employed on several diseases. Hence, for a drug utilized against different cancers, they may require disease-specific sensitivity thresholds.

Two-dimension models currently offer the easiest path to clinical implementation of CSRA. This is due to both their adaptability to various clinical laboratory settings, and shorter turnaround time. However, the diversity of approaches overviewed here offers much promise to generate more complex and more predictive models. Continued integration of live cell culture technologies within clinical laboratories should facilitate their routine use. Moreover, technological advances, especially in image analysis and high content screening 133,186, allow to combine an array of endpoints. This allows better monitoring complex biological processes, either at the individual cell phenotype or tissue architecture level. This will undoubtedly be key to definitely bridge bench and bedside, by offering clinically useful functional assays.

## Regulatory considerations and quality management of functional assays

CDx and CoDx may directly influence a patient's course of treatment. Consequently, they fall under strict regulation, and their manufacturing and execution require a stringent quality management framework. In our introduction, we pointed out CDx and CoDx have received, respectively, official and unofficial definitions. Functional assays, because of their nature, definitely are CoDx: they recommend specific treatment among the available list against a given indication; the medical team ultimately decide the best strategy to apply, based on this information but also on pathology's characteristics and overall patient's status. In addition, functional assays predict treatment response concomitantly for several drug classes, contrary to conventional CDx or CoDx; they may thus require a specific definition. In any case, the regulatory framework applicable to CDx also applies to functional assays.

Regulations in Europe and USA differ in the way they consider CDx as a whole, and functional assays dedicated to treatment individualization in particular. In the US, CDx fall within “class III” products, presenting a potential, unreasonable risk of illness or injury. As such, they require clinical investigation under an Investigational Device Exemption (IDE) before premarket approval (PMA). PMA is issued by the FDA's Center for Devices and Radiobiological Health (CDRH). Approval from the FDA allows commercialisation of CDx, which must be performed in an environment certified under the Clinical Laboratory Improvement Amendments (CLIA). Historically, some functional assays have started as laboratory developed tests (LDT) in clinical institutions 187,188. However, the FDA noted this may lead to medical use of products with unproven performance and insufficient manufacturing controls. Guidance from the FDA issued in 2014 now gives LDT a more traditional validation track to ultimately allow their use in “*making medical treatment decision*” 189. Manufacturers are required to notify the FDA and provide “*basic information*” about their product.

Marketing of MD in the European Union is conditioned to CE marking. In that respect, IVD-MD used to fall under the 98/79 EC directive. It allowed manufacturers to self-certify their diagnostics products for CE marking. Also, CDx were considered as “low-risk devices”, which, given their destination, was not appropriate 6. IVD-MD are now delimited by the 2017/746 regulation, coming into full enforcement in May 2022. It places CDx into “class C” devices, presenting “high personal risk” and “moderate to low risk” to public health. This regulation entails manufacturers to produce assays complying with a set of harmonized requirements regarding their performances. Assays are also expected to be supported by an appropriate QMS. Most recent ISO standards specifically applicable to functional assays as IVD-MD are ISO 13485:2016 (general requirements of QMS for regulatory purposes) and ISO 14971:2012 (risk management). In 2018, the FDA announced its intent to modernize the quality system regulations for medical devices. This decision includes a transition away from 21 CFR 820 towards the ISO 13485 standards. The proposed rule has yet to be issued. It will create opportunities to harmonize global practices in the medical device industry. Although differences are considered minor, the transition for companies is expected to stretch over a few years, following a thorough gap analysis. Assessment of CE compliance is performed by notified bodies (NB). European NBs are currently restructuring to comply with the new regulation.

Inherent to CDx and CoDx is the dependency of risks and benefits of the selected treatment(s) upon the intrinsic performances of the assays. Rigorous demonstration of the assay robustness, reproducibility and clinical benefit must be conducted to achieve sufficient reliability. The three pillars of assay validation are: analytical validation, clinical validation and clinical utility 12. Analytical validation rests on several parameters: repeatability (agreement between successive measures of the same sample), reproducibility (agreement between measures of several samples of the same measurand), precision (proximity of measurement results to the true value), accuracy (degree of repeatability of measurements) and limit of detection (*e.g.* minimal quantity of material to assess for producing a reliable result). In most advanced functional assays, patient's clinical response acts as the gold standard for benchmarking the patient's cell *in vitro* response. Hence, most of the aforementioned parameters must be derived from studies directly involving patients. There is no common rule regarding reproducibility of biomarker assays as a whole; however, coefficients of variation inferior to 15 % were cited as an aim to achieve 190. Accuracy can be studied in early development phases on readily available *in vitro* models such as cells lines 191. Thorough standard operating procedures (SOP), adequate training of operators, quality of assay material (batch control of consumables and reagents, restriction to standardized reagents with an entirely controlled formulation 192), proper instrument controls and maintenance, are other factors that positively influence reproducibility. If not mandatory per se, automation (liquid handling, endpoint measurement) is highly desirable to ensure a throughput compatible with sample turnover at a clinical diagnostics scale, as well as robustness and reproducibility of the whole procedure, thus facilitating compliance with regulatory requirements. For 3D models grown in matrices, liquid handling remains challenging, hence limiting the adaptability of such approaches 133. Laboratories currently offering functional testing services rarely communicate on the technical performances of their technology: Helomics published the positive impact of automation on their workflow for ChemoFX^®^, with improved accuracy and precision 191.

Sample collection, processing and logistics are key steps in the functional assay flowchart, since such tests necessarily deal with live cells or cell extracts 171. Specific media and procedures may be developed to ensure standardization, and thus control, of the whole process 38-40,192. Sample quality depends on available material; it should contain enough tumour tissue, while undesirable zones (necrotic, fibrous, adipose, mucinous, or containing blood clots) are reduced to minimum. The major hurdle in logistics is clearly the time frame between sampling at bedside and processing at the IVD laboratory. Our repeated observations show that: (i) maximum delay for preserved sample integrity is 72 hours; (ii) best sample integrity is obtained with samples processed no later than 48 hours following sampling; (iii) sample cryopreservation is undesirable, as it both lowers the number of available cells to perform the assay and attenuates their response to cytotoxic drugs (unpublished data from our group). This constrained time frame prevents sample shipping across long distances and borders because of obvious technical and legal hazards. It either limits technologies to a domestic market or requires their direct implantation in target countries. It also extends the number of operating days of the laboratory to six per week, to ensure Friday despatch is possible 38-40.

Following proper design controls and performance measurements, clinical validity of the IVD-MD is assessed. It aims at demonstrating its ability to “*identify, measure, or predict the presence or absence of a medical condition or predisposition*” for which the device is intended 12. Key parameters such as diagnostic sensitivity (proportion of positive patients correctly identified as such), diagnostic specificity (proportion of negative patients correctly identified as such), positive predictive value (PPV; proportion of assay-positive patients that are actually positive) and negative predictive value (NPV; proportion of assay-negative patients that are actually negative) are measured at this stage 193,194. For functional assays, endpoint cut-off is crucial, as it is the limit between predicted responders and non-responders to a specific drug. Integration of assay's constraints, especially regarding sampling and logistics, should also be fully evaluated at that stage. Finally, clinical utility is the demonstration that assay's results improve the therapeutic benefits patients obtain from treatment personalization versus a more systematic use. In accordance with the latest recommendations of the ASCO 15, this parameter requires large, randomized, prospective clinical trials investigating patient's outcome (response rates, progression-free survival, or, even better, overall survival at appropriate timepoint) after assay-directed treatment or oncologist-chosen treatment. The results of such trials are yet to be published. Their design is not trivial, as multi-drug regimens make it difficult to gain an insight into the effect of individual drug components.

As previously discussed, the proliferation of experimental or clinically implemented chemosensitivity assays directed against a large array of indications has been accompanied with the development of greatly varying protocols. Although understandable from a technological differentiation standpoint, the major pitfall of such situation is already illustrated by anti-PD-1/PD-L1 antibodies: each marketed antibody has its own CDx, with clinical trials constructed in such a way that they lead to drug-specific cut-offs to declare efficacy 6. Assays cannot be substituted to one another. They require laboratories to be able to perform the whole array of tests to adapt to a physician's preference for one drug or the other. Still, it may be envisioned that ICI CDx will be amenable to interchangeability, because they rely on a common endpoint 195. Because of their extreme technical heterogeneity, however, this will not be possible for functional assays. It may ultimately happen that, for a given pathology, the best predictive model wins it all.

## Concluding remarks

As stated in a founding review 12, CSRA are a significant player in next-generation functional diagnostics, a concept that should not be solely identified with genomics. Indeed, molecular data come in huge volumes and are intricate. Their interpretation is frequently impaired by the difficulty of relating a patient's genotype with the actual behaviour of their tumour. And even with their genome or transcriptome deciphered, few patients will benefit from an already approved molecularly targeted drug 196. In addition, insight into off-label use has been disappointing 197. Identifying the right treatment thus necessitates the exploration of a different level of biological information. The armamentarium already available to predict tumour responses indeed encompasses complementary technologies (high-throughput genomics, immunoprofiling, functional testing) whose concomitant use may provide clinicians with a complete, multilevel profile for each patient, including particular susceptibilities to drug toxicity 198. Such comprehensive data could allow a completely personalized approach in deciding which therapies would best treat each cancer. In that context, CSRA should keep their role as CoDx, prioritizing drug combinations rather than selecting or disqualifying them. Several groups have come to this conclusion 14,179 and some companies include this perspective into their business model.

The main criticism raised against CSRA is the lack of translatability of *ex vivo* response into clinical response 14,199. Despite the recent level of evidence not being considered sufficient to recommend a routine clinical use yet, there is a growing body of cues to show it is actually possible to predict a patient's response to several classes of drugs. Most importantly, this information is useful to improve their chances for prolonged survival and a better quality of life. Besides improved response rates and survival, there are other benefits to be gained from CSRA: (i) reduction in deleterious side effects, due to shortened therapeutic cycles or the avoidance of inefficient drugs; (ii) decrease in costs of global care, due to optimized patient management and a reduced use of expensive drugs.

The intense development of targeted therapies might have caused some to forget that chemotherapies still stand as standards-of-care against cancer. Obviously, their response rates remain insufficient. Use of these “historical” anticancer treatments would benefit from personalized approaches, too, which has been the foundation of CSRA. Failure of chemoresistance prediction now allows efforts to concentrate of chemosensitivity prediction, which clearly holds a much higher clinical utility 199.

While this may remain a pious wish because of industrial reasons, the recent experience gained from the development of “me-too” drugs and CDx in immuno-oncology, with thresholds that are not comparable from one assay to the other while they are supposed to measure the same endpoint, leads us to suggest the creation of working groups and workshops dedicated to functional assays. They would gather the main actors in the field to discuss technical (especially endpoints) and regulatory considerations to help structuring assays.

Personalized medicine offers the perspective of tailoring therapeutic approaches for every patient. Yet, not every patient harbours an already known actionable drug target. Blending biological knowledge with technological development to better grasp the behaviour of every patient's cancer should ensure that none is left in the ditch along the road.

## Author Contributions

Pr. M. Mathonnet, Dr. C. Bounaix Morand du Puch, Dr. N. Christou, Dr. S. Giraud and Dr. C. Lautrette conceived the original idea. Dr. C. Bounaix Morand du Puch and Dr. M. Vanderstraete drafted the manuscript with equal contributions. Pr. M. Mathonnet revised the manuscript.

## Figures and Tables

**Table 1:** Overview of main chemosensitivity assays developed over the past five decades

Functional assay	Culture method	Endpoint(s)	First study	Ref
Clonogenic assay	3-D matrix culture of tumour cells	Colony formation and counting	1970s	17
DiSC	Cell monolayer	Counting of cell mortality using light microscopy	1983	19
HDRA	3D (collagen sponge)	MTT/[^3^H] thymidine incorporation	1986	23
FCA	Tumour fragments	Esterase-driven formation of fluorescein	1988	27
EDRA	Tumour fragments	[^3^H] thymidine incorporation	1990	21
MiCK	Cell monolayer	Measure of cell apoptosis by spectrophotometry	1994	30-34
CD-DST	3D (collagen droplets)	MTT/ATP bioluminescence	1996	26
ATP-CRA	Cell monolayer	ATP bioluminescence	1997	22
ChemoFx^®^	Cell monolayer	Counting of cell number by fluorescence microscopy	2002	36
The Oncogramme^®^	Cell monolayer	Counting of cell mortality using light microscopy	2010	38-40
ChemoID^®^	Cell monolayer	Measure of cell proliferation using WST-8	2014	35
CANScript^TM^	Tumour fragments	Multiple	2015	37

**Table 2:** Overview of the analytical performances of the major chemosensitivity assays described in the literature against a large array of solid cancers

Assay	Cancer	Success rate	Accuracy	PPV	NPV	Cohort size (number of patients)	Sensitivity	Specificity	Authors	References
ATP	Gastrointestinal	85.0%	84.0%	-	-	25	64.0%	100.0%	Kawamura et al., 1997	41
ATP	Ovary	89.0%	71.0%	66.0%	89.0%	93	95.0%	44.0%	Konecny et al., 2000	42
ATP	Ovary	85.0%	85.0%	50.0%	100.0%	33	100.0%	82.0%	Ng TY et al., 2000	43
ATP	Ovary	-	70.7%	83.0%	56.5%	161	68.8%	74.3%	O'Meara et al., 2001	44
ATP	Lung	90.6%	90.0%	100.0%	80.0%	31	83.3%	100.0%	Kim BS et al., 2004	45
ATP	Lung	90.6%	-	-	-	53	-	-	Kang et al., 2005	22
ATP	Lung	43.8%	68.8%	61.1%	78.6%	34	-	-	Moon YW et al., 2007	46
ATP	Breast	93.0%	85.0%	100.0%	66.7%	43	78.6%	100.0%	Kim et al., 2008	47
ATP	Ovary	69.0%	90.0%	94.1%	-	29	94.1%	-	Han et al., 2008	48
ATP	Gastrointestinal	95.8%	77.8%	85.7%	75.9%	36	46.2%	95.7%	Kim JH et al., 2010	49
ATP	Colon	79.0%	-	94.0%	38.0%	62	-	-	Lee et al., 2011	50
ATP	Bladder	96.3%	74.3%	83.7%	66.7%	54	97.6%	20.0%	Ge et al., 2012	51
ATP	Ovary	-	-	83.0%	84.8%	80	88.6%	77.8%	Zhang et al., 2015	52
HDRA	Head and Neck	88.0%	74.0%	83.0%	64.0%	26	71.0%	78.0%	Robbins et al., 1994	53
HDRA	Gastric & Colon	96.3%	-	66.7%	100.0%	38	100.0%	90.6%	Furukawa et al., 1995	54
HDRA	Ovary	97.0%	87.0%	88.0%	86.0%	15	88.0%	86.0%	Ohie et al., 2000	55
HDRA	Breast	98.8%	80.0%	100.0%	70.0%	15	62.5%	100.0%	Tanino H et al., 2001	56
HDRA	Head and Neck	97.6%	-	-	-	42	-	-	Singh et al., 2002	57
HDRA	Head and Neck	-	91.7%	90.0%	100.0%	19	79.0%	66.7%	Ariyoshi et al., 2003	58
HDRA	Head and neck	-	77.8%	76.9%	80.0%	49	90.9%	57.1%	Hasegawa et al., 2007	59
HDRA	Head and Neck	91.0%	74.0%	69.0%	80.0%	57	79.0%	71.0%	Pathak et al., 2007	60
HDRA	Lung	97.4%	83.0%	73.2%	100.0%	343	100.0%	68.1%	Yoshimasu et al., 2007	61
HDRA	Ovary	-	-	62.0%	81.0%	61	90.0%	43.0%	Neubauer et al., 2008	62
HDRA	Oesophagus	89.3%	-	-	-	53	66.7%	55.6%-66.7%	Fujita et al., 2009	63
HDRA	Glioma	94.0%	-	-	-	33	100.0%	60.0%	Gwak H et al., 2011	64
HDRA	Colon	-	66.3%	-	-	86	72.7%	54.7%	Yoon et al., 2012	65
ITRA	Colon	-	61.9%	57.1%	64.3%	42	44.4%	75.0%	Yoon et al., 2017	24
ITRA	Ovary	-	44.4%	40.0%	66.7%	18	85.7%	18.2%	Kim et al., 2019	25
CD-DST	Multiple	80.0%	91.0%	80.0%	100.0%	11	100.0%	86.0%	Kobayashi et al., 1997	66
CD-DST	Breast	84.3%	87-94.4%	83.3%	95.5-100%	70	92.9%	62.5-95.5%	Takamura et al., 2002	67
CD-DST	Mesothelioma	-	50.0%	-	-	26	100.0%	36.0%	Higashiyama et al., 2008	68
CD-DST	NSCLC	-	70.0%	50.0%	92.0%	81	88.0%	63.0%	Higashiyama et al., 2010	69
CD-DST	Gastric	80.0%	-	-	-	64	-	-	Naitoh et al., 2014	70
CD-DST	OSCC	81.8%	92.3%	90.9%	100.0%	14	-	-	Sakuma et al., 2017	71
FCA	Multiple	-	-	85.0%	97.0%	73	98.0%	81.0%	Leone et al., 1991	27
The Oncogramme^®^	Colorectal	97.4%	63.6%	64.7%	60.0%	19	84.6%	33.3%	Bounaix Morand du Puch et al., 2016	39
CANScript™	Multiple	-	-	93.9%	100.0%	55	-	-	Majumder et al., 2015	37
ChemoFX^®^	Ovary	-	-	63.6%	100.0%	18	-	-	Ness et al., 2002	72
ChemoFX^®^	Breast	83.9%	-	-	-	62	-	-	Mi et al., 2008	73
ChemoFX^®^	Head and Neck	72.7%	-	81.8%	-	22	-	-	Jamal et al., 2017	74
ChemoID^®^	Glioblastoma	-	-	54.6%	100.0%	11	100.0%	50.0%	Claudio et al., 2017	75
MICK	Endometrium	78.9%	-	-	-	19	-	-	Ballard et al., 2010	32
Mean		86.6%	76.1%	76.7%	82.0%	51.8	84.2%	68.3%		
Median		89.2%	77.8%	82.4%	81.0%	40.0	88.0%	72.7%		

**Table 3:** Meta-analysis of studies that explored through clinical investigation the capacity of chemosensitivity assays in improving patient outcomes

Reference	Authors	Pathology	Assay	Cohort size	Randomisation	Treatment	Outcomes
76	Strickland et al., 2013	AML	MiCK	109	No	According to physicians	MiCK assay results correlate well with clinical outcome of patients in terms of OS and response rate.
67	Takamura et al., 2002	Breast	CD-DST	70	No	According to physicians	No differences in OS between drug-sensitive and resistant patients.Longer TTP in drug-sensitive patients (15.6 *vs*. 2.5 months, p < 0.005).
77	Bosserman et al., 2015	Breast	MiCK	30	No	CSRAs results to be used at physician's discretion	The use of the MiCK assay led to a higher response rate (38.1% *vs*. 0%, p = 0.04), and longer TTP (7.4 *vs*. 2.2 months, p < 0.01).
78	Kim et al., 2014	Breast	HDRA	50	No	According to physicians	No correlation between breast cancer subtype and chemoresponse found using HDRA.
79	Shinden et al., 2016	Breast	HDRA		No	Paclitaxel	Paclitaxel inhibition rate is significantly associated with DFS (p = 0.036).
80	Mekata et al., 2013	Colon	CD-DST	151	No	According to physicians	No differences in OS for patients found with high- and low-sensitivity for 5-FU.Significant differences in 5-year RFS (p = 0.04).
81	Ji et al., 2017	Colon	HDRA	89	No	5-FU	Better 5-year PFS in chemosensitive group.No significant improvement of OS.
82	Hur et al., 2012	Colorectal liver metastasis	CD-DST	63	Yes	According to CSRA results or physician's choice	Better treatment response.
83	Kubota et al., 1995	Gastric cancer	HDRA	128	No	Mitomycin C and tegafur	OS and DFS are longer in the HDRA-sensitive group for both drugs.
70	Naitoh et al., 2014	Gastric cancer	CD-DST	64	No	According to CSRA results	Higher survival rate in patients found as drug sensitive (p = 0.019). Longer time to progression (p = 0.023).
84	Tanigawa et al., 2016	Gastric cancer	CD-DST	206	No	S-1	Better relapse-free survival in drug-responder subgroup (p = 0.0014).
85	Howard et al., 2017	Glioblastoma	ChemoID^®^	41	No	According to physicians	Longer OS and recurrence time in patients with positive stem cell chemoprofile.
57	Singh et al., 2002	Head and Neck	HDRA	41	No	5-FU, cisplatin	Correlation between HDRA chemoresponse and clinical outcome.
86	Wilbur et al., 1992	Lung	DiSC	45	No	According to physicians	Improved OS in drug-sensitive patient subgroup (p = 0.04).
46	Moon et al., 2009	Lung	ATP	120	No	CSRA-guided treatment	No significant differences in PFS and OS between both groups.Higher response rate in ATP subgroup (71% *vs*. 38%, p = 0.023).
87	Akazawa et al., 2017	Lung	CD-DST	39	No	platinum-based adjuvant chemotherapy	Better 5-year DFS in chemotherapy-sensitive patients (p = 0.037).No differences in OS.
88	Inoue et al., 2017	Lung	CD-DST	87	No	According to phyisicians	No differences in OS and 5-year DFS.
89	Chen et al., 2017	Lung	ATP	120	No	According to physicians	Improved PFS and OS in chemosensitive groups (p = 0.046 and p = 0.041, respectively).
90	Ugurel et al., 2006	Melanoma	ATP	53	¨No	Assay-directed chemotherapy	Chemosensitive patients showed improved OS (14.6 months *vs*. 7.4, p = 0.041). Progression arrest in more patients (59.1% *vs*. 22.6%, p = 0.01).
33	Bosserman et al., 2012	Multiple	MiCK	40	No	CSRAs results to be used at physician's discretion	Increased response rates when physicians used MICK assay (44% *vs*. 6.7%, p > 0.02).
91	Kurbacher et al., 1998	Ovary	ATP	55	No	According to CSRA results	Higher overall response rate (64% *vs*. 37%, p = 0.04) in Assay group.Better PFS in platinum-refractory patients (p = 0.004).
92	Gallion et al., 2006	Ovary	ChemoFX^®^	256	No	According to physicians	Correlation of ChemoFX assay results with Progression-Free Interval.
93	Cree et al., 2007	Ovary	ATP	147	Yes	According to CSRA results or physician's choice	No significant differences for OS, RR or PFS.
94	Herzog et al., 2010	Ovary	ChemoFX^®^	192	No	According to physicians	Correlation of ChemoFX assay results with median OS.
34	Salom et al., 2012	Ovary	MiCK	150	No	According to physicians	Longer OS and RFP in stage III and IV patients that received the best chemotherapy (p > 0.01 and p = 0.03, respectively).
95	Jung et al., 2013	Ovary	HDRA	104	No	According to physicians	Longer PFS in chemosensitive patients (34.0 *vs*. 16.0 months, p = 0.03).
96	Park et al., 2016	Pancreas	ATP	57	No	Gemcitabine	Better disease-free survival in gemcitabine-sensitive patients (p = 0.017)

## Abbreviations

2D: two-dimension
3D: three-dimension
ASCO: American Society of Clinical Oncology
CAF: cancer-associated fibroblast
CAM: chick chorioallantoic membrane
CDRH: Center for Devices and Radiobiological Health
CDx: companion diagnostics
cGMP: current good manufacturing practices
CLIA: Clinical Laboratory Improvement Amendments
CSRA: chemosensitivity and resistance assay
CoDx: complementary diagnostics
CRC: colorectal cancer
CRO: contract research organisation
EMA: European Medicines Agency
FDA: Food and Drug Administration
ICI: immune checkpoint inhibitor
HIPEC: intraperitoneal chemotherapy associated to hyperthermia
IVD: *in vitro* diagnostics
IVD-MD: *in vitro* diagnostics medical device
LDT: laboratory developed tests
MD: medical device
MoA: mode of action
MSI: microsatellite instability
NB: notified body
NPV: negative predictive value
OS: overall survival
PBL: peripheral blood lymphocyte
PDX: patient-derived xenograft
PFS: progression-free survival
PIPAC: pressurized intraperitoneal aerosol chemotherapy
PMA: Premarket Approval
PPV: positive predictive value
QMS: quality management system
RECIST: response evaluation criteria in solid tumours
SOP: standard operating procedures
TIL: tumour-infiltrating lymphocyte
TMB: tumour mutation burden
TME: tumour microenvironment
